# The Carbon Footprint of Finishing Yearling Bulls Fed a Diet Containing Vegetable By-Products in Navarra, Spain

**DOI:** 10.3390/ani16162576

**Published:** 2026-08-18

**Authors:** Pablo González-Martínez, Irantzu Goenaga, Sara León-Ecay, José Antonio Mendizabal, Noelia Aldai, Kizkitza Insausti, Maite M. Aldaya

**Affiliations:** 1 ETSIAB Escuela Técnica Superior de Ingeniería Agronómica y Biociencias, ISFOOD Universidad Publica de Navarra, 31006 Pamplona, Spain; pablo.g.m@outlook.com (P.G.-M.); igoenaga@trasa.es (I.G.); sara.leon@unavarra.es (S.L.-E.); jamendi@unavarra.es (J.A.M.); maite.aldaya@unavarra.es (M.M.A.); 2 Tratamiento Subproductos Agroalimentarios, S.L. (TRASA), Camino San Juan s/n, 31320 Milagro, Spain; 3 Lactiker Research Group, Department of Pharmacy and Food Sciences, Universidad del País Vasco/Euskal Herriko Unibertsitatea, 01006 Vitoria-Gasteiz, Spain; noelia.aldai@ehu.eus

**Keywords:** carbon footprint, vegetable by-products, livestock production, beef, circular economy

## Abstract

Livestock farming is blamed for its significant carbon footprint (CF), contributing to environmental pollution and climate change. Among other approaches, this has highlighted the need to find alternative feeding systems for cattle production that are potentially able to reduce greenhouse gas (GHG) emissions. In this context, the objective of the present study was to compare the CF of producing cattle fed a Conventional diet versus cattle fed a diet that included vegetable by-products (VBP diet) sourced from the local agri-food industry. In this study, twenty-four entire male young bulls were reared in Navarra, Spain. Twelve calves were finished on the VBP diet that also included fodder and grain, and the remaining animals were finished with a local Conventional diet based on concentrate and straw. Results showed a larger CF of meat from animals fed the Conventional diet in comparison with the VBP-fed ones, that is, 117.84 kg versus 42.01 kg of CO_2_ equivalent per kilogram of meat, respectively. This research demonstrates that using by-products from the local agri-food industry for feeding cattle has an important beneficial effect on the environment. It is not only a circular economy solution that recovers and recycles by-products instead of treating them as waste, but can also notably reduce the GHG associated with livestock production. This study marks the beginning of ongoing research into the effect of introducing regional vegetable by-products in the diet of livestock and their corresponding impacts on productivity and the environment, particularly in terms of GHG emissions per kilogram of feed consumed.

## Simple Summary

Livestock farming has been criticized for producing large amounts of greenhouse gases that contribute to climate change. This study investigated whether feeding cattle with vegetable by-products from local food-processing industries could reduce the environmental impact of beef production compared with a Conventional diet. Twenty-four young bulls were raised in Spain, with half receiving a diet that included vegetable by-products and the other half fed a Conventional diet. The results showed that meat from cattle fed vegetable by-products had a much lower carbon footprint, producing about 42 kg of Carbon Dioxide Equivalent per kilogram of meat compared with nearly 118 kg for cattle fed a Conventional diet. The study concludes that using vegetable by-products as cattle feed can significantly reduce emissions while also preventing useful materials from becoming waste. This approach supports a circular economy and could help make food production more environmentally sustainable, benefiting society by reducing the environmental impact of livestock farming.

## 1. Introduction

Greenhouse gas emissions are considered an environmental issue [1] regarding their effect on the global warming of the Earth. Anthropogenic GHG emissions have continued growing in recent decades [1] as a result of human activity and population growth [2]. Moreover, one-third of the food that is produced for human use gets lost or wasted globally, amounting to approximately 1.3 billion tons per year [3]. Food loss occurs through all the different food supply chain steps. This considerable amount of food that gets wasted and lost indicates that the food system is inefficient, being detrimental to food security, the environment, producer profits, consumer prices, and increasing climate change [4].

The Food and Agriculture Organization of the United Nations (FAO) estimates that agricultural emissions reached 10.7 GtCO_2_e year^−1^ in 2019, while the Intergovernmental Panel on Climate Change (IPCC) Special Report on Climate Change and Land [5] estimates them at 12.0 GtCO_2_e year^−1^. Excluding land use-related emissions from total agricultural activities, considering only emissions up to the farm gate, FAO estimates that in 2019 the total reached 7.2 GtCO_2_e year^−1^, with the main source being livestock emissions, responsible for 51.4% of these emissions (including enteric fermentation and manure emissions) [6].

In general terms, the livestock sector has been estimated to account for 14.5% of global GHG emissions [7]. Within the sector, beef production contributes the majority of emissions, producing 41% of the livestock sector’s direct emissions [8].

In the context of the environmental impact of beef cattle feeding practices, a notable deficiency in the literature is identified, both globally and nationally, regarding the utilization of by-products from the local agri-food industry. Among the limited studies available, the investigation on the use of distillery by-products in livestock feed stands out, emphasizing that, in terms of GHG emissions, this practice proves more sustainable than energy production through biogas [9]. In other species, it has also been questioned whether industry co-products could reduce environmental impact in pig production, for example [10].

Feeding strategies based on grazed pasture have also been investigated in beef cattle production. In contrast to the limited information available on the use of agro-industrial by-products as feed ingredients, a substantial body of research has examined the effects of pasture-based systems on animal performance and environmental outcomes [11,12]. These studies have shown that pasture-based feeding generally requires lower external energy inputs than conventional feedlot systems. However, cattle raised on pasture often require a longer production period to reach slaughter weight than animals finished in more intensive systems. This longer production cycle can increase resource use and greenhouse gas emissions per kilogram of meat produced. These trade-offs, together with the limited knowledge regarding the incorporation of agri-food industry by-products into beef cattle diets, highlight the need for a more comprehensive evaluation of feeding practices in beef production systems.

Another study, very similar to the topic of the present article, was carried out in Spain at the University of Cordoba, using by-products from the local agri-food industry to fatten calves for meat production [13], but the focus was put on the acceptability and quality label preferences by Spanish meat consumers. This study from southern Spain showed that fattening calves on a diet rich in fibrous local agro-industrial by-products inedible to humans improves the colour, flavour and tenderness of the meat, increasing its acceptability to meat consumers. It has also been demonstrated to be a strategic way to reduce the impact of the meat industry on the environment and reduce competition for food between livestock and humans, while lowering production costs and dependence on raw materials for meat production. In this context, the term “vegetal by-product” encompasses plant/vegetable by-products, which are co- or by-products originating from processing industries or losses within the food production chain for human consumption [14]. In the realm of plant processing, particularly fruits and vegetables, substantial by-products are generated due to size standards and processing criteria. Notably, the vegetable processing industry generates substantial amounts of organic residues. For every tonne of harvested vegetables, approximately 600 kg is converted into marketable products, whereas the remaining 400 kg, including roots, leaves, stems, and other non-marketable plant fractions, becomes organic residue or by-product [15].

The concept of a CF is intrinsically linked to the amount of carbon dioxide (CO_2_) emissions associated with all the activities of a person or entity, encompassing everything from buildings and corporations to countries. It includes direct emissions and those generated to produce the electricity associated with consumed goods and services. Moreover, the CF concept often incorporates emissions of other GHGs, such as methane, nitrous oxide, or chlorofluorocarbons (CFCs).

Annually, the world food supply chain generates 13.7 billion metric tons of carbon dioxide equivalents (CO_2_ eq.) [16]. This represents 26% of total anthropogenic GHG emissions. A significant increase in food chain-related emissions is anticipated as population and income levels rise. Therefore, in line with IPCC guidelines, reducing the emissions of the food supply chain is critical [16].

To go one step forward on the state of the art, the present study was designed to compare two diets for beef fattening from an environmental impact perspective. Building upon a prior study where the water footprint was assessed [17], the focus now shifts towards quantifying greenhouse gas emissions using the CF. The aim is thus to compare a diet formulated primarily based on vegetable by-products of the local agri-food industry, known as the “VBP diet,” with a “Conventional diet” commonly used in northern Spain, based on concentrate and straw. First of all, the description of the case study and system boundaries is provided, and secondly, the CF of one kg of beef produced under the European Quality Scheme *Ternera de Navarra* Protected Geographical Indication (PGI) has been assessed for each of the diets studied. Then, the results have been compared with the existing literature in this field. In this context, the present research confirms these findings and opens an opportunity for improving feeding management and making livestock systems more sustainable.

## 2. Materials and Methods

### 2.1. Case of Study and System Boundaries

#### 2.1.1. System Boundaries

In this study, a CF assessment was conducted on two groups of calves. The assessment compared beef finished under two different feeding regimes. The first group was fed a Conventional diet consisting of concentrate and straw during the fattening phase. This diet was chosen because it is a common practice in Spain [18], and it represents high-input, globally sourced feed. The second group was fed with an alternative diet based on VBP from the local agri-food industry. The experimental diets were formulated to be isoproteic, with both the Conventional and VBP diets containing 126 g crude protein/kg dry matter (DM). However, a slight difference in energy content was observed, with the Conventional diet providing 19.1 MJ net energy/kg DM compared with 18.8 MJ/kg DM for the VBP diet. During the 5-month finishing period, animals fed the Conventional diet consumed an average of 1200 kg feed per animal, whereas those receiving the VBP diet consumed 1800 kg per animal. In addition, all animals underwent a 2-month pre-fattening transition period during which feed intake averaged 600 kg per animal. Productive performance differed between diets, as carcass yield was significantly higher in animals fed the Conventional diet (62.9%) than in those receiving the VBP diet (61.8%) (*p* = 0.013).

**Figure 1:**
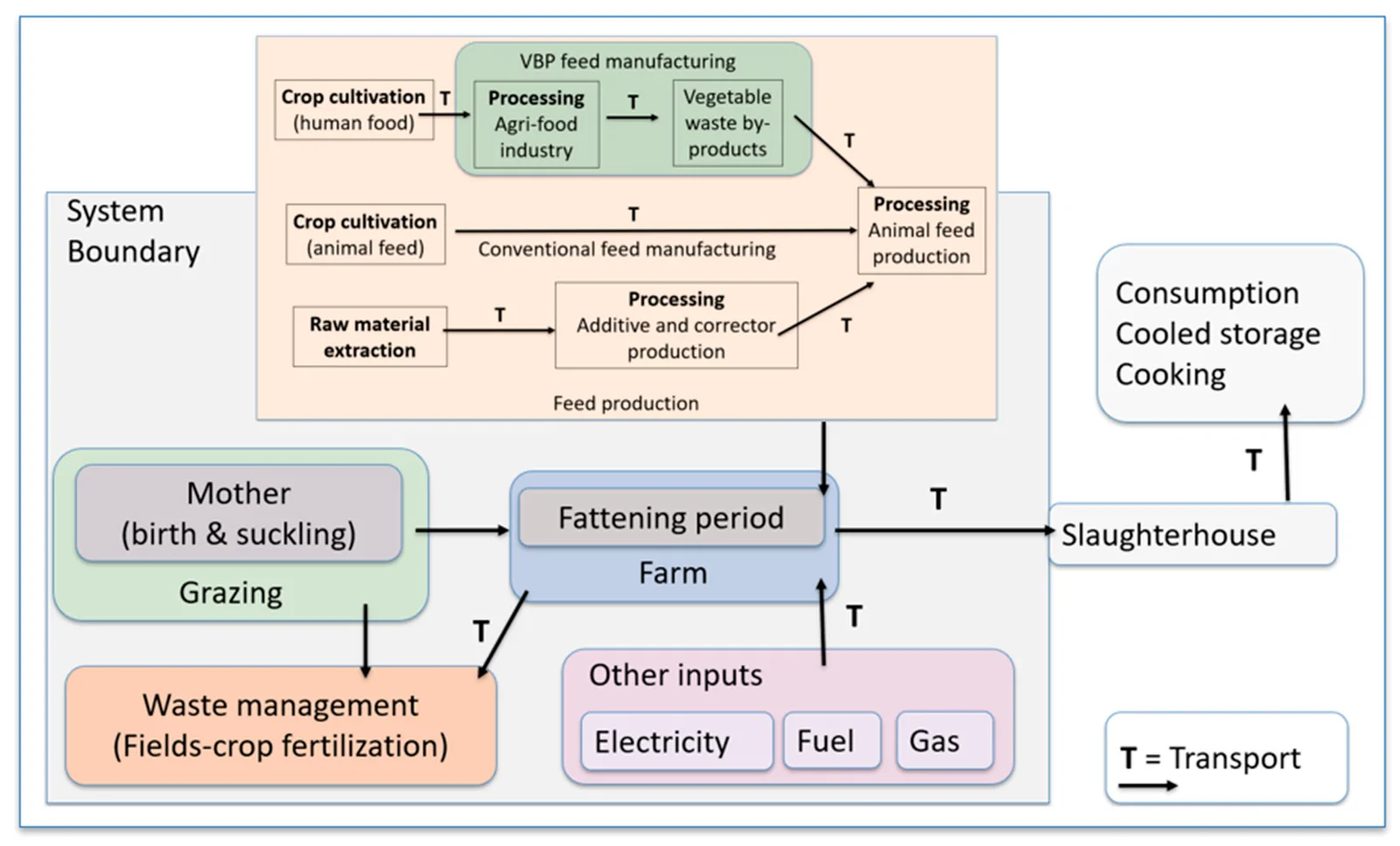
System boundary of the two beef cattle feeding regimes under study: Conventional and vegetable by-product-based diets.

The CF assessment spanned from the farm to the slaughterhouse gate (Figure 1). The functional unit was defined as 1 kg of meat. Meat production per animal was calculated from the cold carcass weight and the corresponding carcass yield, and inventory data were subsequently expressed per kilogram of meat produced. Downstream phases, including distribution, consumer use, and disposal, were excluded from the study as they were deemed irrelevant for comparison.

Detailed information on the composition and origin of ingredients can be consulted in Table A1 in Appendix A [17].

The aspects considered for CF calculation were similar in both feeding systems. However, differences related to approach and data availability exist, as explained below. The primary distinction in terms of approach is that VBP production involves a stage not present in conventional production: the generation of vegetable by-products.

#### 2.1.2. Production and Transport of Feed Ingredients

The production and transport of feed ingredients were reported in detail in [17].

In brief, the origin of the products varied. The VBP diet was mainly local, consisting of plant by-products from the local agri-food industry in Navarra. Only 14% of its ingredients were of international origin (3 out of 22) [19]. By contrast, 54% of the ingredients of the Conventional diet were sourced internationally (7 out of 13). In these cases, information on the crop production system or the specific location of crops within each region or country was not available. The information that could be accessed was obtained from the company producing the Conventional diet feed, which provided the country of origin of each ingredient. From there, estimates began to be made from databases such as the USDA to obtain the most productive areas or regions for each ingredient in each country.

In the case of the VBP diet, the vegetable by-products came from the local agri-food industry of the Ebro Valley, Navarra, Spain.

Regarding transport, the environmental impact associated with the transportation of ingredients from their production sites to livestock facilities was assessed. Transportation was considered in both feeding systems, encompassing the movement of raw materials from their origin to the food processing plant, and subsequently, the conveyance of feed from the processing plant to the beef cattle farm. For the calculation of the CF of ingredient transportation, certain assumptions were made based on available evidence when detailed data were lacking.

The production location of each ingredient was obtained from the countries and production areas provided by both animal feed manufacturing companies. When production areas within a supplying country were unknown, such as for forages imported from America or Europe (outside Spain), the likely production regions were identified using USDA Foreign Agricultural Service databases [20] (PSD Database and World Agricultural Production reports), which provide crop production statistics by country and region worldwide.

Regarding transportation, information on the type of vehicle, the most common fuel, average fuel consumption, and the emission factor of that fuel in CO_2_ equivalents were obtained from scientific literature and the Spanish System of Inventory of Emissions (Ministerio de Transición Ecológica, n.d.) 21.

#### 2.1.3. Animal Experimental Design

The animal experiment design was also previously reported in detail [17].

In brief, calves were reared under the requirements of the PGI Ternera de Navarra. During the first 4 months of life, calves suckled their mothers while grazing. Then, a short transition period with concentrate and straw was established before fattening the animals with the experimental diets (from 5 to 13 months of age), as established in the PGI regulations. Until month 5, both batches (Conventional and VBP) received exactly the same management and feeding.

The feeding trial of the two diet regimes under study started at the beginning of December 2021. During the fattening phase, calves were divided into two feeding groups: (a) two pens of 6 animals fed Conventional diets (n = 12) and (b) two pens of 6 animals each fed VBP diet (n = 12). Experimental procedures for livestock management followed the European Directive 2010/63/EU, regulated by Royal Decree 348/2000 in Spain.

The animals were slaughtered at the Almameat S.L. slaughterhouse in Salinas de Pamplona, Navarra, Spain, in accordance with Regulation (EC) No. 1099/2009 on the protection of animals at the time of slaughter (European Union, 2009).

### 2.2. Carbon Footprint Assessment

In this section, the methodology for assessing the CF is detailed using the ISO 14067:2019 standard [22] and the LCA methodology, produced following International Organization for Standardization (ISO) 14040 standards [23]. Elements of PAS 2050:2011 were consulted in specific cases not covered by ISO 14067.

#### 2.2.1. Definitions

Before entering into details on how the CF has been assessed, some definitions are listed to better understand the following sections.

Product Carbon Footprint: As outlined in ISO 14067:2019, the Carbon Footprint of a product (CFP) is the sum of GHG emissions and removals from a product system. These are expressed as the mass of CO_2_e emitted per functional unit, calculated based on the LCA methodology, focusing solely on the unique impact assessment category of climate change.

Greenhouse Gases (GHGs): GHGs are components of the atmosphere, both natural and anthropogenic, that absorb and emit radiation at specific wavelengths within the infrared spectrum emitted by the Earth’s surface, atmosphere, and clouds (ISO 14067:2019).

Global Warming Potential (GWP): GWP is an index, founded on the radiative characteristics of GHGs, which quantifies the radiative forcing subsequent to a pulse emission of one unit mass of a particular GHG in the present atmosphere, integrated across a selected time frame (usually 100 years), in comparison to that of CO_2_ (ISO 14067:2019).

Carbon Dioxide Equivalent (CO_2_e): All greenhouse gas emissions were converted to constant CO_2_e. CO_2_e is a unit used to compare the radiative forcing of a GHG with carbon dioxide. The GWP of 1 for CO_2_, 25 for CH_4_, and 298 for N_2_O is used [5,24,25] (Table 1).

The Life Cycle (LC): LC (ISO 14067:2019) consists of consecutive and interrelated stages related to a product, from the acquisition of raw materials or their generation from natural resources to the final treatment at the end of the product’s life.

Life Cycle Assessment (LCA): LCA is the collection and evaluation of inputs, outputs, and potential environmental impacts of a product system throughout its life cycle (ISO 14067:2019). LCA follows a comprehensive approach, preferably “cradle-to-gate,” aiming to assess resource consumption and environmental and health effects caused by systems and processes necessary to obtain products (goods and services). The LCA begins with the acquisition of raw materials to create the product and ends at the point where materials become waste and are managed as such. The LCA was conducted following IPCC guidelines (2019), considering greenhouse gas emissions, natural resource consumption, and other relevant indicators.

#### 2.2.2. Analysis and Procedures

Once the scope of the system and the functional unit have been defined, and with the system boundaries and diagram designed, the identification of all unit processes that emit GHG in the LCA needs to be done in order to quantify emissions and define the activity data to multiply those units by the corresponding emission factors.

The unit processes that emit GHG are:

- Enteric fermentation
- Manure management
- Direct N_2_O emissions
- Indirect N_2_O emissions
- Ingredient production
- Transportation of ingredients and manufactured feed
- Electricity consumption throughout the process
- Farm to slaughterhouse transport emissions

Emission data were collected based on IPCC, and Tier 1 and 2 methodology were employed for calculations. Information related to transportation has been detailed later in the chapter concerning the transport of ingredients and manufactured feed. Concerning electricity, data were obtained directly from the databases of the Spanish Ministry for Ecological Transition and Demographic Challenge (MITECO). The electricity distributors supplying the feed production facilities and the corresponding electricity consumption associated with the feed produced for the BEEF+ project (Government of Navarra) experiment were individually considered.

Animal respiratory CO_2_ emissions and net emissions of soil C from manure application were assumed to be 0 because animal respiration CO_2_e is not a net source of GHG [26,27].

A custom-built calculator in Microsoft Excel is used to calculate the CF of meat from both diets following the LCA model determined above, from cradle to gate. The system boundaries included the fattening and finishing phases (pasture, cereal straw and feedlot). Animal performance, herd management practices, transport, feed inputs and machinery use were based on empirical data.

##### Enteric Fermentation

The enteric fermentation was calculated using Equation 10.19 of Chapter 10 of the 4th volume of the IPCC [24].

$$Enteric\;fermentation\;emissions\left(\frac{{kg\;CO}_{2}}{kg\;meat}\right)=\frac{EF\times N_{T}\times GWPEFCH4}{meat\;kg/year}$$

where:

- EF = emission factor for the defined livestock population, kg CH_4_ year^−1^
- N = the number of heads of livestock species/category T in the country
- T = species/category of livestock
- GWP EFCH_4_ = 25 for CH_4_

##### Manure Management

The manure management was calculated using Equation 10.23 of Chapter 10 of the 4th volume of the IPCC [24]. The information was taken from the different tables, considering the solid storage system that the farmer used to manage the manure of the cattle before being applied to the different fields once it was sufficiently dry. It was scaled up to an annual basis, 365 days, for inventory purposes.

$$EF\left(\frac{{kg\;CO}_{2}}{kg\;meat}\right)=\left({VS}_{T}*365\right)\left[B_{o\left(T\right)}*\frac{0.67kg}{m^{3}}*\sum \frac{{MCF}_{S,K}}{100}*{MS}_{\left(T,S,K\right)}\right]$$

where:

- EF = emission factor for the defined livestock population, kg CH_4_ year^−1^
- VS_T_ = daily volatile solids excreted for livestock category T, kg dry matter animal^−1^ day^−1^
- 365 = basis for calculating annual VS. production, days year^−1^
- B_o(T)_ = maximum methane-producing capacity for manure produced by livestock category T, m^3^ CH_4_ kg^−1^ of VS. excreted; GWP EF CH_4_ = 25 for CH_4_
- 0.67 = conversion factor of m^3^ CH_4_ to kilograms CH_4_
- MCF_S,k_ = methane conversion factors for each manure management system S by climate region k, %
- MS_(T,S,k)_ = fraction of livestock category T’s manure handled using manure management system S in climate region k, dimensionless. Once the factor emission was obtained using the equation explained above, it was multiplied by the number of animals in the batch and by the GWP EF CH_4_ = 25 for CH_4_, so the result is in CO_2_e.

##### Direct N_2_O Emissions

The direct N_2_O emissions were calculated using Equations 10.25 and 10.30 of Chapter 10 of the 4th volume of the IPCC [24]. The information was taken from the different Tables (10.19, 10A-4 and 10.21) as well as from primary data such as the average weight of the animals from each batch or the solid storage system that the farmer used to manage the manure of the cattle.

$$N_{2}O_{D\left(mm\right)}=\left[\sum_{S}\left[{\sum_{T}\left(N_{\left(T\right)}{Nex}_{\left(T\right)}{MS}_{\left(T,S\right)}\right)}_{2}\right]{EF}_{3\left(S\right)}\right]*\frac{44}{28}$$

where:

- N_2_O_D(mm)_ = direct N_2_O emissions from Manure Management in the country, kg N_2_O year^−1^
- N_(T)_ = number of heads of livestock species/category T in the country
- Nex_(T)_ = annual average N excretion per head of species/category T in the country, kg N animal^−1^ year^−1^
- MS_(T,S)_ = fraction of total annual nitrogen excretion for each livestock species/category T that is managed in manure management system S in the country, dimensionless
- EF_3(S)_ = emission factor for direct N_2_O emissions from manure management system S in the country, kg N_2_O-N/kg N in manure management system S
- S = manure management system
- T = species/category of livestock
- 44/28 = conversion of (N_2_O-N)(mm) emissions to N_2_O(mm) emissions
- Nex _(T)_ = N_(rate (T)) TAM/1000 * 365

where:

- Nex_(T)_ = annual N excretion for livestock category T, kg N animal^−1^ year^−1^
- Nrate(T) = default N excretion rate, kg N (1000 kg animal mass)^−1^ day^−1^
- TAM(T) = typical animal mass for livestock category T, kg animal^−1^

Once the N_2_O_D (mm)_ is computed, a conversion to weight of kg of CO_2_e multiplying by 298 is done, so that it can be converted from kg CO_2_e/year to kg CO_2_e/kg meat.

##### Indirect N_2_O Emissions

The indirect N_2_O emissions were calculated using Equations 10.28 and 10.29 of Chapter 10 of the 4th volume of the IPCC [24]. The information was taken from the different Tables (10.19, 10A-4,10.21 and 10.22) as well as from primary data such as the average weight of the animals from each batch or the solid storage system that the farmer used to manage the manure of the cattle.

$$N_{leaching-MMS}=\sum_{S}\left[\sum_{T}\left[\left(N_{\left(T\right)}{Nex}_{\left(T\right)}{MS}_{\left(T,S\right)}\right)*{\left(\frac{{Frac}_{leachMS}}{100}\right)}_{\left(T,S\right)}\right]\right]$$

where:

- N _leaching-MMS_ = amount of manure nitrogen that leached from manure management systems, kg N year^−1^
- N_(T)_ = number of heads of livestock species/category T in the country
- Nex_(T)_ = annual average N excretion per head of species/category T in the country, kg N animal^−1^ year^−1^
- MS_(T,S)_ = fraction of total annual nitrogen excretion for each livestock species/category T that is managed in manure management system S in the country, dimensionless
- Frac _leach MS_ = percent of managed manure nitrogen losses
- N_2_O_(L(mm)) = (N_(leaching-MMS) [EF]_5) * 44/28

where:

- N_2_O L(mm) = indirect N_2_O emissions due to leaching and runoff from Manure Management in the country, kg N_2_O year^−1^
- EF5 = emission factor for N_2_O emissions from nitrogen leaching and runoff, kg N_2_O-N/kg N leached and runoff (default value 0.0075 kg N_2_O-N (kg N leaching/runoff)^−1^.

Once the N_2_O L(mm) is computed, a conversion to the weight of kg of CO_2_e, multiplying by 298, is made; afterwards, another conversion from the weight of kg of CO_2_e/year to kg CO_2_e/kg meat is done.

##### Ingredient Production

The calculation of the ingredient production CF can be divided into 3 different components. The first one has to do with the calf suckling phase. In this period of four to five months, the calves consume the milk of their mothers while they stay in the pastures close to the farm. This period lasts 130 days. The following period goes from the sixth to the eighth months of life of the animals, during which all calves were fed on the Conventional diet. The differences between the two feeding regimes began from the 8th month onwards until slaughter. In this third period, 12 of the calves were fed on a Conventional diet, whereas the other 12 were fed on the VBP diet.

To calculate the weight of kg of CO_2_e emitted during the first stage, the quantity of milk consumed by the calves was estimated. Calves were assumed to gain approximately 10% of their live weight during this stage, and the corresponding milk intake was calculated accordingly. Once the amount of milk consumed was determined, it was multiplied by the emission factor (EF) associated with the dam’s milk. This EF was obtained from a previous study [28], which considered diets for lactating cows containing more than 30% corn. In addition, greenhouse gas emissions generated during lactation were allocated between milk production and calf growth.

For the rest of the calculations to compute the CF of the ingredients, in the second and third periods of the feeding of the calves, the French databases of Agrybalise and INRAE were consulted to obtain the EF of the different ingredients. The calculation consists of multiplying the amount of feed of each of the ingredients of the diet by the period that each type of diet lasts, by the FEF of each ingredient. Then, all ingredient emissions are added, and this number is divided by the meat produced from an animal in a calendar year (which is obtained considering the cold carcass weight and the carcass yield). Once this is known, it is multiplied by the emission factor of each ingredient so that the emissions in kg CO_2_e/year are calculated.

##### Transportation of Ingredients and Manufactured Feed

In order to estimate the CF related to transport, the activity unit (kilometres travelled by each vehicle) is multiplied by the EF of each fuel type [24,29]. To get the kg CO_2_e/kg of meat, the kg CO_2_e of transport is divided between the meat produced with each of the different feeds. The meat/year was calculated from the mean of the last weight estimates done before the cattle was transported to the slaughterhouse and multiplying this mass by the average carcass yield of the different feed groups. Once the average meat yield per animal was determined, it was converted from the 13-month duration of the entire process into a natural (12-month) year. In this way, all the calculations have the same units.

In the case of the Conventional diet, as 7 out of 13 ingredients were sourced internationally, not only trucks were needed to transport the products, but also bulk carriers. For the VBP diet, only the TRASA S.L. company’s truck was used.

$$Transport\;Emissions=\sum \frac{D_{\left(VT\right)}*Fuel\;EF}{\frac{meat}{year}}$$

where:

- *D*_(*VT*)_ = Distance travelled with each type of vehicle used for each type of feed (Conventional and VBP)
- Fuel EF = emission factor for the different types of fuel used by the vehicles at different moments of the ingredient transportation
- Meat/year = the total amount of meat produced in a natural year by each of the groups under experiment

##### Electricity Consumption Throughout the Process

The electricity consumption considered in the meat production belongs to the feed manufacturing process. The rest of the electricity consumed is considered to be negligible. The cowshed is illuminated with daylight, and the electricity consumed in the office was minimal.

The activity data of the electricity consumed in the feed production process in KWh/ton is multiplied by the EF of the electricity marketer and then divided by all the meat produced by each of the different batches under study.

$$Electricity\;Emissions=\sum \frac{{Activity\;data}_{\left(kWh\right)}*Electricity\;company’s\;EF}{\frac{meat}{year}}$$

where:

- Activity data _(kWh)_ = Energy used to produce all the feed used during the fattening period for each batch
- Electricity company’s EF = emission factor for the different type of energy production system used by the electricity supplier to provide the electricity
- Meat/year = the total amount of meat produced in a natural year by each of the groups under experiment

##### Farm-To Slaughterhouse Transport Emissions

A total of 5 return trips were made to the slaughterhouse. An average of 4.8 calves from the experiment were transported on each trip. According to data sources [30], the trucks transporting live animals weighing 525 kg have a capacity of 28 animals. Therefore, as each trip also transports animals that did not belong to the project, we imputed the kilometres travelled according to the ratio of animals in the experiment versus occupied places. In this way, the kilometres travelled by the liveweight in kilograms of calves under study were obtained, and this data was multiplied by the emission factor of diesel, the fuel used in this type of heavy vehicle weighing more than 3.5 t (Ministerio de Transición Ecológica, n.d.) [21]. Once the kg CO_2_e is obtained, it is divided by the total amount of meat to obtain the emissions per kg of meat.

#### 2.2.3. Vegetable By-Products Allocation Procedure

The challenge of most agri-food production systems is the partitioning of environmental impacts among by-products. The problem is that different allocation approaches and methods lead to different results. Here, to allocate the environmental impact of vegetables to their by-products used for animal feed, three of the most commonly used and acknowledged allocation systems were used and compared. Thus, we ensured that the CF meat results from the comparison of the Conventional and VBP diets were accurate and reliable. In relation to the Conventional diet, some by-products (e.g., soy hulls) are also used. Emission factor data for soy hulls were obtained directly from the literature, as access to precise data was not available for their calculation (product and mass percentage of by-products, and economic value).

First, a physical allocation was applied, which assigns an allocation factor using physical characteristics, that is, the mass associated with each by-product. In this case, the allocation factor is defined as the quantity of the output product obtained per quantity of input product. Nevertheless, the physical allocation could be considered inadequate for the lower-valued by-products.

To prevent this unfairness, the economic allocation was used. That is, the market price of the product and by-products was used to calculate the allocation factors. In the economic allocation, the inputs and outputs of the process or system are divided between respective economic values of its products.

Finally, physical and economic criteria were used together to assign environmental loads to co-products and by-products. In order to calculate this allocation, the VBP WF allocation methodology was consulted. The Water Footprint Assessment Manual [31] suggests a very objective way of evaluating the VBP, incorporating in the same formula the physical proportions of each VBP and its economic weight. The water footprint allocation method of the Water Footprint Assessment Manual has been applied to the CF field. The calculations for this allocation are as detailed in the following equation:

$${CF}_{prod}\left[P\right]={(CF}_{proc}\left[P\right]+\sum_{i=1}^{y}\frac{{CF}_{prod}\left[i\right]}{f_{p}\left[P,i\right]})xf_{v}\left[P\right]$$

in which CF_prod_[P] is the CF of output product p, CF_prod_[i] is the CF of input product i and CF_proc_[P] is the process CF of the processing step that transforms the y input products into the z output products, expressed in carbon emissions per unit of processed product p. Parameter f_p_[P,i] is a so-called “product fraction” (physical allocation) and parameter f_v_[P] is a “value fraction” (economic allocation).

The weights for the physical allocation were calculated taking into account the percentages of by-products generated by the local agri-food industry in the Ebro valley, and provided by the TRASA company (Table 2). The market value of the VBP, considering depreciation of machinery, transporters’ salaries, vehicle costs, etc., has been considered; the price for the VBP was estimated at 13.5 €/ton. This information was also provided by TRASA. The market value (national average prices) of the crops was found in the Spanish Ministry of Agriculture, Fisheries and Food [32].

The physic-economic allocation is the one used for comparison with the Conventional diet. It includes both physical and economic considerations, so it is the most complete to evaluate the VBP.

### 2.3. Animal Ethics Statement

The study was conducted on a commercial farm and complied with all applicable animal welfare regulations and standard livestock management practices. As no additional experimental interventions were imposed on the animals beyond routine farm management, ethical committee approval was not required.

## 3. Results

In this section, the different allocations related to the vegetable by-products used in the VBP diet are presented first. Following, the study’s results are categorized by GHG-emitting stage. Subsequently, a comparative analysis of emissions across various activities, including on-farm emissions such as enteric fermentation, manure management, and N_2_O emissions, is provided. Next, emissions from ingredients grouped by type are presented, concluding with emissions categorized by carbon emission indicators.

### 3.1. Effect of the Different Allocation Systems on the Carbon Footprint

The results that the allocations have affected are only those linked to the production of ingredients. More specifically, they are associated with the production of feed for the VBP diet. All other inputs and calculations have been kept constant for each of the three allocations. In relation to the Conventional diet, some by-products (e.g., soy hulls) are also used. Emission factor data for soy hulls were obtained directly from the literature, as precise data were not available for their calculation (product and mass percentage of by-products and their economic value).

The results obtained (Table 3) after applying the allocation systems described in Section 2 dedicated to this concept are 1.56, 1.52 and 1.53 kg CO_2_e/kg meat/year for the physical, economic and physic-economic allocations, respectively. These results, together with the remaining sections common to all of them, account for 42.01, 41.97, and 41.97 kg CO_2_e/kg meat/year, respectively. All three results are lower than the CF emissions from conventional feed ingredients presented in Table 4.

The physic-economic allocation has been the allocation chosen to represent the results of the VBP diet. It is the most complete allocation in the calculation, as it includes both the mass of the VBP per product and its economic valuation.

### 3.2. Carbon Footprint of 1 kg of Meat

In evaluating the overall CF for the production of 1 kg of meat (functional unit) over a year, a comparison between the Conventional and VBP diets reveals distinct environmental footprints. The Conventional diet demonstrated a total CF of 117.84 kg CO_2_e/kg meat/year, whereas the VBP diet exhibited a notably lower impact at 42.01 kg CO_2_e/kg meat/year, estimated using a physical-economic allocation.

Even if the total CF is bigger for the Conventional diet, six out of the nine GHG-emitting activities (Table 4) considered in the CF calculation have higher emissions for each kg of meat produced by the animals fed the VBP diet compared to animals fed the Conventional diet.

### 3.3. Comparison of the GHG Emissions by Activity of the Conventional and Vegetable By-Product-Based Diet

**Figure 2:**
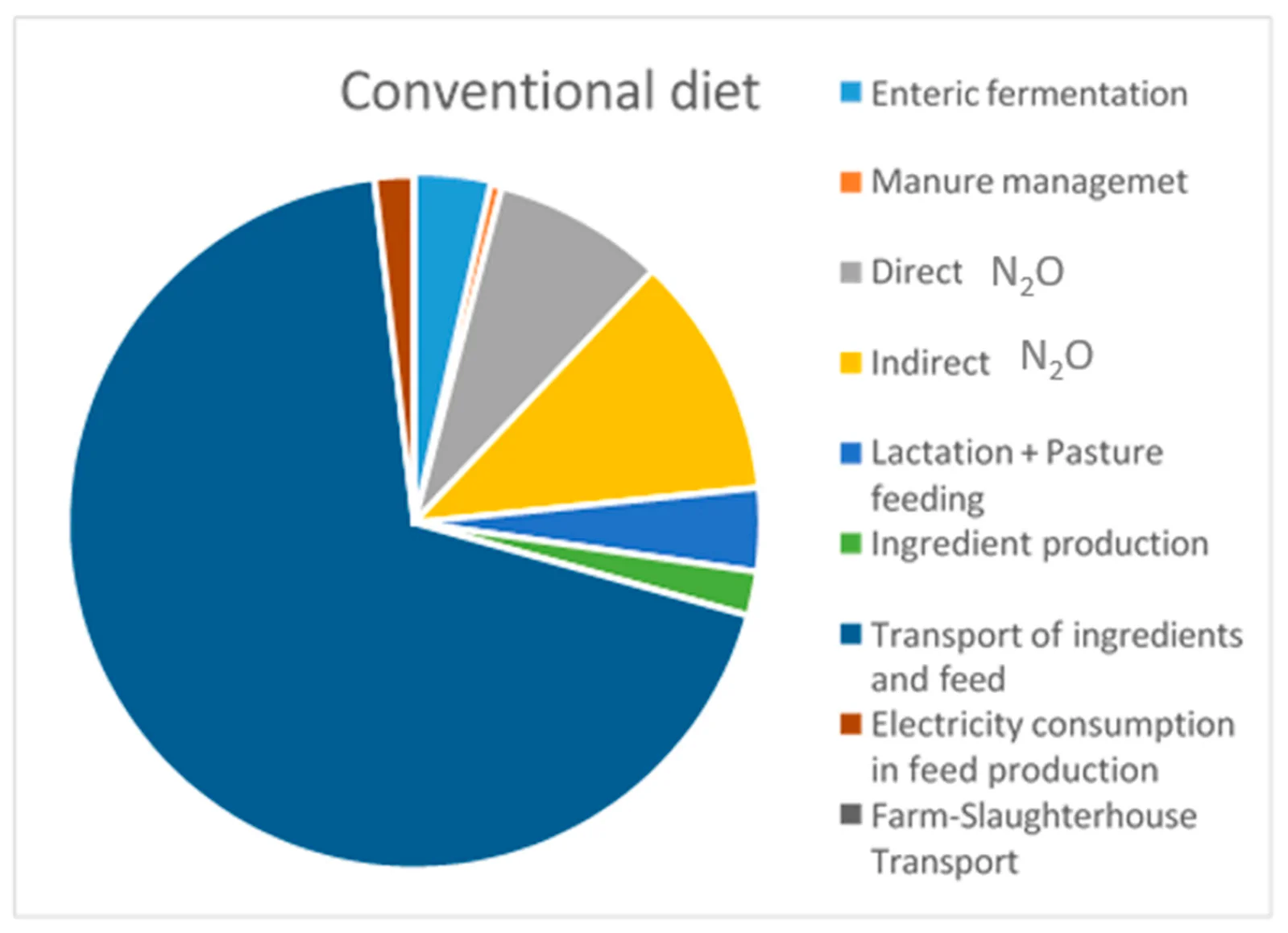
Conventional diet GHG activity contribution to total CF.

A detailed analysis of GHG emissions by activity further elucidates the specific contributors to the different CF calculated for the Conventional and VBP diets (Table 4). In the Conventional diet (Figure 2), transport of ingredients was calculated to be 81.12 kg CO_2_e/kg meat/year, and the indirect and direct N_2_O emissions were 13.33 and 9.42 kg CO_2_e/kg meat/year, respectively. Both emerged as the primary sources of emissions.

**Figure 3:**
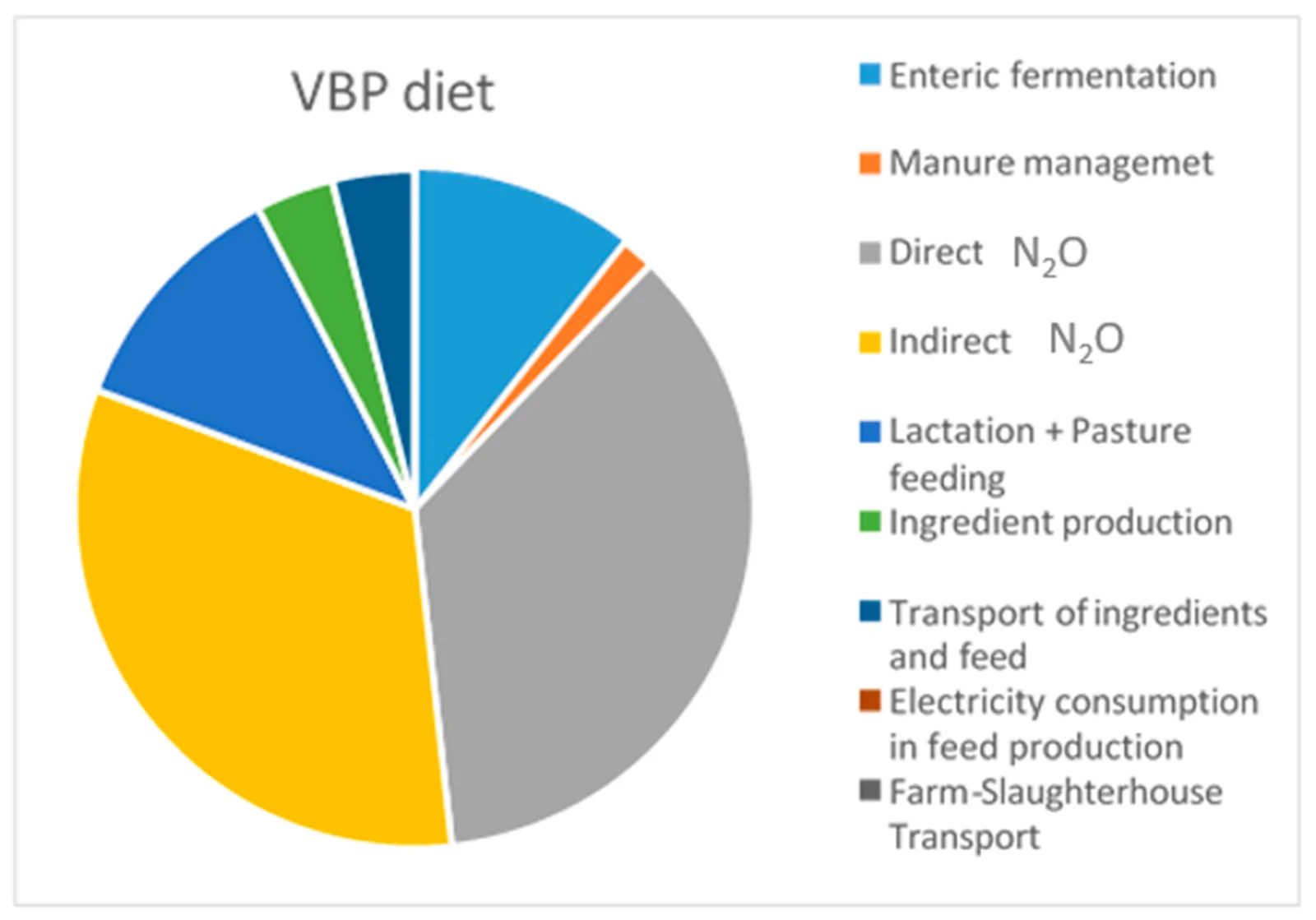
VBP diet GHG activity contribution to total CF.

Conversely, the VBP diet (Figure 3) exhibited elevated emissions from direct N_2_O emissions (15.15 kg CO_2_e/kg meat/year) and indirect N_2_O emissions (13.63 kg CO_2_e/kg meat/year).

The Conventional diet feed ingredient production was calculated to be 2.05% of the total CF, whereas the VBP feed was calculated to be 1.32%. The bigger difference between the activities emitting GHG emissions, in terms of percentage of the total CF, was the transport of the ingredients, which was calculated to be 69% in the Conventional diet and 1% in the VBP diet.

### 3.4. Farm GHG Emissions

In Table A2, Table A3, Table A4 and Table A5 of Appendix A, the baseline information and the calculator of the activities contributing to the total emissions in kg CO_2_e in each diet are provided. The four of them (enteric fermentation, manure management, direct and indirect N_2_O emissions) show that the starting data is the same, as they are data obtained from the same source [21,24].

The subtle disparities observed in the results of the four processes documented in this section stem from variations in data-processing methods, transforming the emissions per activity from the weight of kg of CO_2_e to kg CO_2_e/kg of meat. These differences are primarily attributed to variations in animal live weight between batches, as well as disparities in carcass yield. Specifically, the batch with the higher carcass yield (63% for Conventional compared to 62% for VBP diets), which in this case is the batch from animals fed the Conventional diet, ultimately demonstrates superior performance per kg of meat produced.

### 3.5. Ingredients Production and Emissions

**Figure 4:**
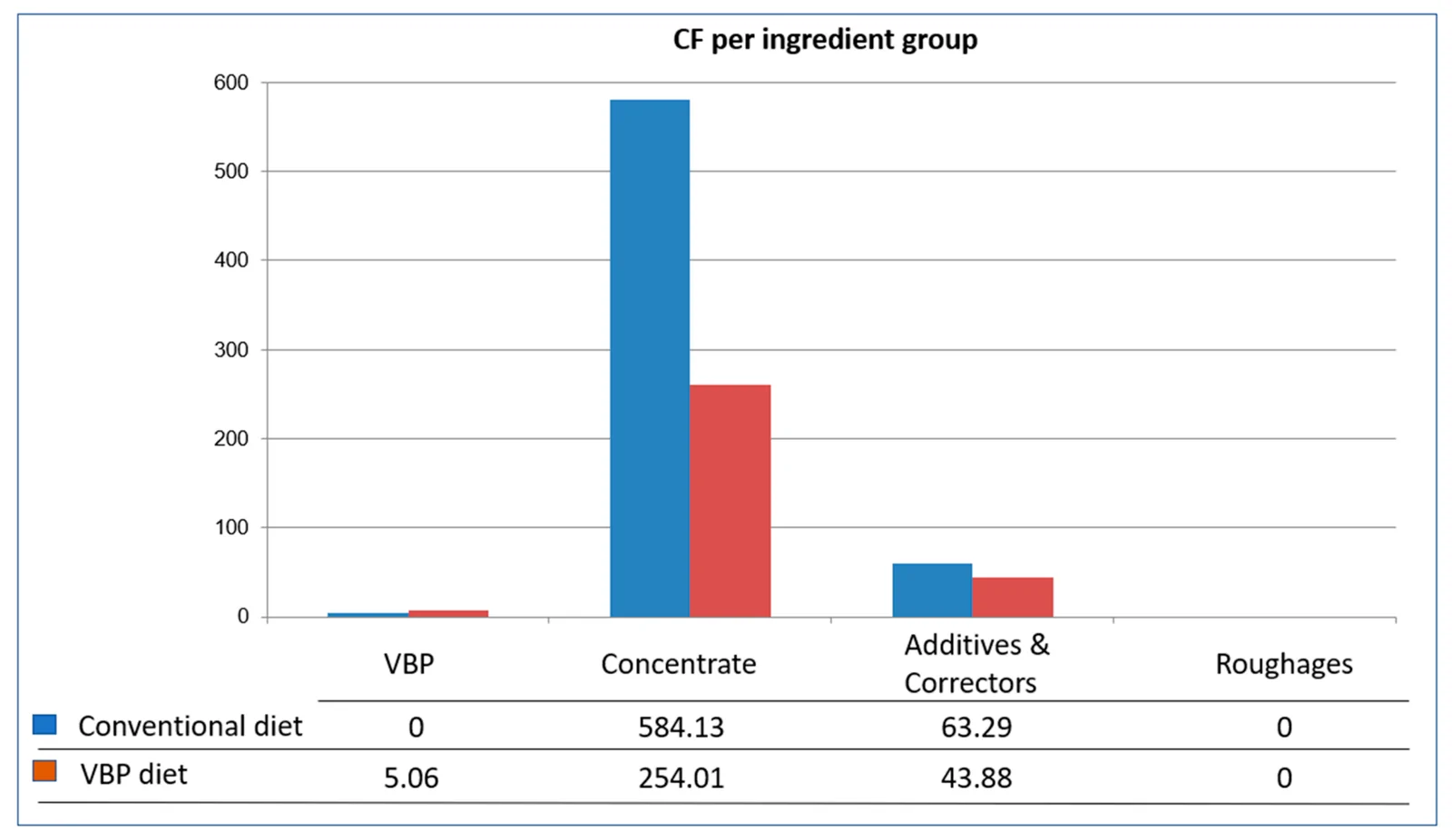
CF per ingredient group used to fabricate the diets under study: Conventional and VBP diets.

If the transport from the original production place of the ingredients to the manufacturing plant is kept separate, there is a slight difference in the carbon used to produce one kg of meat from a Conventional diet and the VBP diet. The difference in the ingredient production is 0.9 kg CO_2_e per animal (Table 4) and is mainly found in the production of the ingredients before they are processed in the manufacturing plant. The biggest difference in terms of CF between the two cattle diets is related to the difference in the percentage of concentrate per feed mix in each of the rations. The remaining differences among ingredient families were not substantial enough to be considered meaningful. Only emissions from the ingredients have been considered to build Figure 4, keeping transport and electricity separate. The VBP have a low incidence in the total computation, as do the straws, which also have very low emissions as they are a by-product of cereal production.

### 3.6. Nutritional Indicators for Carbon Emissions Productivity

**Figure 5:**
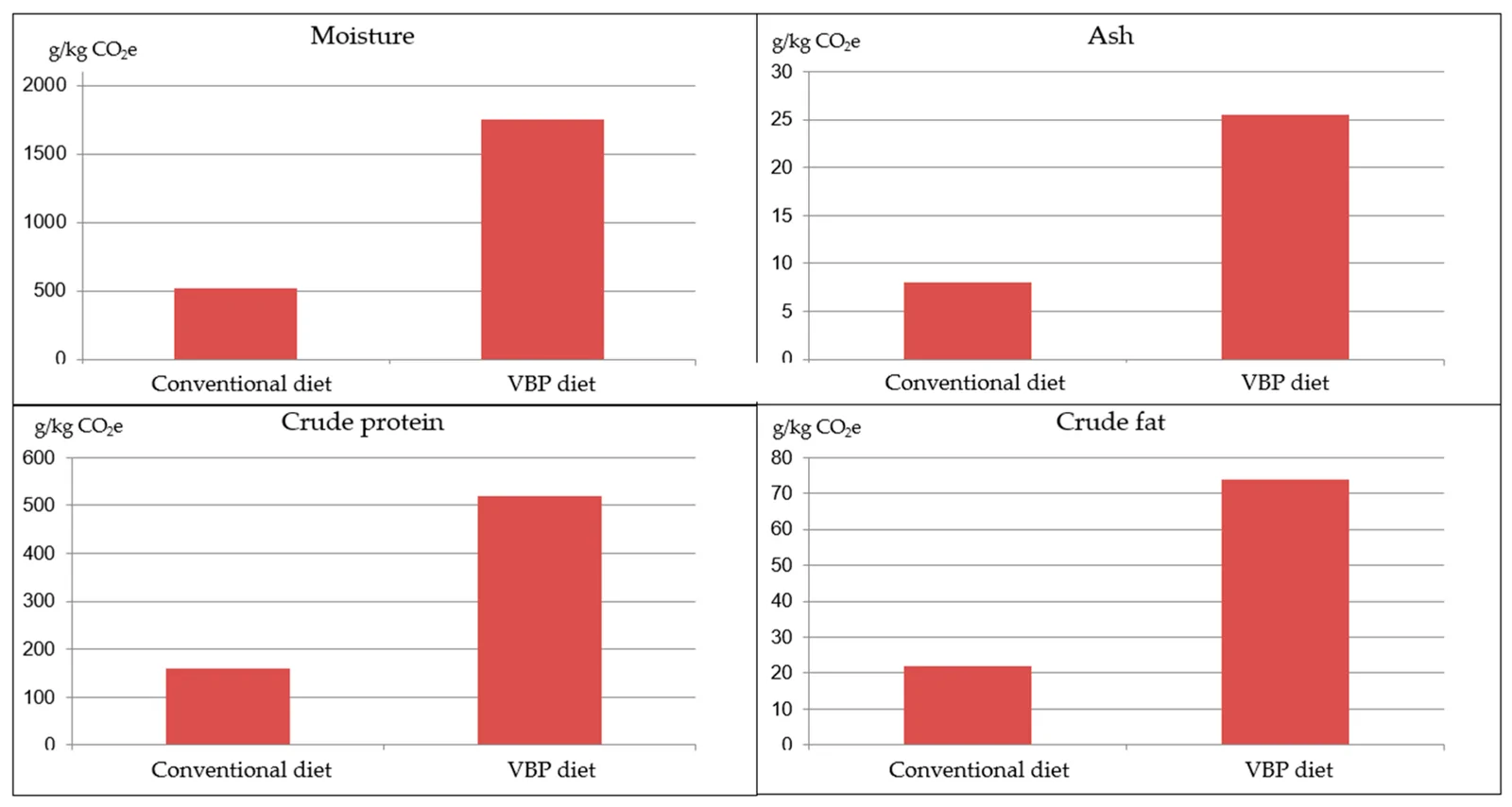
Carbon productivity, in g/kg CO_2_e, for crude protein, crude fat, moisture and ash for young bulls fed a Conventional diet and a VBP diet.

Figure 5 compares the amounts of moisture, ash, crude protein, and crude fat obtained per kg of CO_2_e emitted during the production of meat and bones from young bulls reared under the *Ternera de Navarra* PGI and fed two different diets: a Conventional diet and a VBP diet.

For all the nutritional indicators, the VBP diet has higher quantities of crude protein, crude fat, moisture and ash per kg of CO_2_e emissions (Figure 5).

## 4. Discussion

Previous findings [33] indicate that American and European cattle feeding systems are generally highly efficient due to time and feed intensification based on corn and cereal grains. In recent years, this management approach has become more efficient, producing higher percentages of meat globally. Efficiency is also said to be driven by an increase in carcass yield; the more meat per animal, the smaller the CF per functional unit. This is exactly what has happened in this study, with six of the nine GHG-emitting activities being lower in the yearling bulls fed a Conventional diet, due to a difference of 1% in the carcass yield (63% vs. 62%). Except for ingredient transport, ingredient production and electricity, VBP-fed calves showed a higher CF in every other GHG-emitting activity. This discrepancy occurred because those sections shared the data baseline, as it was collected following the Tier 1 method of the IPCC guidelines [5].

Nevertheless, the overall CF was higher in animals finished under the Conventional diet. Notably, the Conventional diet was significantly disadvantaged in ingredient production. This was expected, considering the difference in the concentrate percentage between diets (87% vs. 53% for Conventional and VBP, respectively). This situation benefits the VBP diet in two ways: not only does it have a lower percentage of concentrate, which has a higher FE, but the VBP, thanks to the by-product physic-economic allocation applied based on the opportunity cost, results in significantly lower CO_2_ emissions per kilogram of meat.

Finally, related to ingredient production, one of the aspects that also makes the footprints of the two diets different was the transport of some of these raw materials to the manufacturing facilities. It emerges as a critical factor, with Conventional diet ingredients sourced from distant countries contributing to a notable difference of 79.5 kg of CO_2_ equivalent per kilogram of meat per year. The VBP diet is made up entirely of products of European origin, and to a large extent from Navarre or the surrounding area. The conventional diet, on the other hand, brings in raw materials, such as soya or palm oil, from the American continent that together made up more than 10% of the feed composition, and from north-western European countries. As a consequence, the Conventional diet had higher CF use per kg of meat. This aggregation, together with the VBP CF due to its allocation, clearly explains the difference in ingredient production between the two diets.

Regarding the allocation methods for the calculation of the CF of the VBP diet, the physical allocation may favour or disadvantage one or more by-products depending on the ratio of by-product generation by characteristics, factory rules, or consumer preferences. This makes it less equitable for all by-products than economic allocation, which treats all by-products uniformly, since in this case TRASA S.L. transports and processes them collectively. Thus, the value assigned for the calculation of the CF with the physic-economic allocation is equal for all VBPs.

Notably, the VBP diet demonstrated a higher efficiency value regarding nutritional indicators, achieving better rates in grams per kilogram of CO_2_ produced in all studied aspects (moisture, ash, crude protein, and crude fat). This means that the lower the CF, the higher the rates per kg of CO_2_ emitted. Therefore, these values can be increased as long as there is room for improvement in the reduction in the footprint, either by improving the efficiency of carcass yields [34], reducing the duration of fattening to reach the desired kg of live weight for slaughter [35], reducing emissions from enteric fermentation due to genetic improvement [36], or type of feed, promoting better degradability of fibres, etc.

Comparing the results of CF in cattle from different studies is a difficult task for many reasons. There exists a lack of consistency in terms of the boundaries chosen for the studies. For instance, in most regions, the beef and dairy sectors are linked, as surplus dairy calves are finished for beef production and culled dairy cows also provide meat. However, the allocation of the environmental burdens between the beef and dairy sectors for many of these cases is inconsistently performed [37]. Another difficulty when making comparisons between studies is the inconsistent inclusion of emission sources. For instance, calculations rarely include GHG emissions associated with capital goods, such as the manufacturing of farm machinery. Further, the impact of land-use change and land-management change on soil carbon is frequently not considered in the CF, but if it is, there are significant challenges to determine the appropriate amortization period and spatial extent associated with the emissions from land-use change [38]. Moreover, the inclusion of the respiratory CO_2_ of cattle and the soil organic carbon (SOC) sequestration by crops, pasture, or linked with other practices, including tilling, is sometimes calculated and others assumed to be minimal and therefore not considered [26,27]. In this study, it was considered to be 0, as data were not available for the location of the croplands. This concept is undoubtedly one of the options that have been increasingly considered as solutions to the challenges of livestock farming and agriculture in achieving a more resilient system against climate change by sequestering atmospheric carbon in soil deposits [39]. As more is known about the subject and as long as there is soil capacity to sequester carbon, there are options for reducing the CF of meat production.

As a result, most of the CF estimates associated with beef production reported in the literature are limited in some way, making comparison between studies challenging.

A comparison with previous studies [33] can be seen in Table 5. In their study, a comparison was made of the CF from calves from the major beef-producing countries and those with the highest percentage of cattle globally were compared, excluding some large countries due to lack of data. This data source has been interesting to compare with our study, despite being a few years old, because it has helped us to see that our results are well above all the results included. This fact opens the door to drawing conclusions and analyzing the reasons for such differences in kg of CO_2_e.

Potential reasons for these differences in the weight of kg of CO_2_e include transportation assumptions and higher direct and indirect N_2_O emissions, which comprise a larger percentage of the total CF than in other studies. Transportation also emerges as a significant contributor to the elevated CF in our study. Assumptions made during transportation calculations, despite thorough research into distances travelled, transportation modes, and fuel types, may lack the desired precision. Optimizing the accuracy of these calculations could potentially narrow the gap observed in CF values. In other studies, transport contribution to the total emissions is less relevant. A noteworthy factor contributing to the disparity is the substantial contribution of direct and indirect nitrous oxide (N_2_O) emissions in our study. This discrepancy aligns with other research [25], emphasizing the need for a more nuanced approach to estimating N_2_O emissions.

Previous findings [27,34] reveal notable discrepancies in the proportions of GHG-emitting activities considered in CF assessments compared to our study. In this sense, some researchers reported that enteric fermentation contributes to 26.7% of the total CO_2_e emissions, while N_2_O emissions contribute 16.5%, ingredient production accounts for 47.9%, and transportation accounts for 4.8% of the total [27]. Others [34] used higher baseline data averages for comparative purposes, reporting CF values of 30.8 and 29.2 kg CO_2_ equivalent per kilogram of carcass. The latter study also indicated higher percentages for enteric fermentation emissions compared to ours, approximately 46% of total emissions. Moreover, it was also stated that these emissions are primarily influenced by variations in feed quality [40].

In addition, the scope of the study is very important; it can be seen that most of them have been carried out by combining a Tier 1 with a Tier 2. This is valued as a very positive practice because it allows for much more specific and precise calculations. In this study, the level of access to the data has been the most basic; almost all of it has been taken from regional or national databases, with exceptions such as French databases for the emission factors of the ingredients. This approach to the study is undoubtedly one that will change in the future, as it is a major limitation. In this study, there were no other options for obtaining more precise data.

In summary, the results indicate that CF assessments in cattle farming are highly sensitive to methodological choices, data availability, and assumptions regarding transportation, feed sourcing, and N_2_O emissions. The limitations encountered in this study, particularly the lack of detailed information on ingredient origins, manure management, and soil carbon dynamics, contributed to uncertainty in the estimates and may explain some of the discrepancies observed when compared with other studies. These findings highlight the importance of comprehensive data collection and standardized assessment methodologies to improve the accuracy, reliability, and comparability of environmental impact evaluations in livestock production systems.

## 5. Conclusions

This study highlights the importance of adopting a holistic and methodologically robust approach to evaluating the CF of beef production systems. Beyond the characteristics of the feeding strategy itself, factors such as system boundaries, data quality, allocation methods, and supply chain transparency can substantially influence environmental assessments and the conclusions drawn from them. Consequently, improving methodological consistency and access to high-quality data is essential for generating reliable and comparable CF estimates.

From a practical perspective, the incorporation of regional vegetable by-products into cattle diets represents a promising pathway towards more circular and resource-efficient livestock production systems. By valorizing materials that would otherwise have limited use, these feeding strategies have the potential to contribute to more sustainable agricultural supply chains while reducing dependence on conventional feed resources. However, environmental performance should be evaluated alongside productive efficiency to ensure that sustainability gains are achieved without compromising overall system effectiveness.

Future research should focus on refining CF methodologies through improved data acquisition, more accurate characterization of emissions sources, and the development of transparent allocation frameworks. Further investigation into the relationship between feed utilization, animal performance, and GHG emissions will also be necessary to fully understand the long-term sustainability of vegetable by-product-based feeding systems. Ultimately, advancing these areas of research will support evidence-based decision-making and help determine the role of alternative feed resources in the transition towards more sustainable beef production.

## Figures and Tables

**Table 1:** Carbon Dioxide Equivalent conversion to represent potential of global warming [25].

GHG	Quantity (™)	Quantity in CO_2_e (™)
CO_2_	1	1
CH_4_	1	25
N_2_O	1	298

**Table 2:** Estimated percentages of by-product generation by the local agri-food industry in the Ebro valley, Spain. Source: TRASA.

Vegetable	By-Product Generation%
Broccoli	17
Cauliflower	15
Green bean	11
Pepper	10
Aubergine	4
Pumpkin	4
Potato	3
Carrot	3
Pea	2
Spinach	2
Red bean	1
White bean	1

**Table 3:** The GHG-emitting activities that contribute to the total carbon footprint of the diet that includes vegetable by-products (VBP) using different allocation systems (physical, economic and physic-economic) (kg CO_2_e/kg meat/year).

VBP Diet			
Allocations	Physical Allocation	Economic Allocation	Physic-Economic Allocation
**GHG-Emitting Activities**			
Enteric fermentation	4.46	4.46	4.46
Manure management	0.67	0.67	0.67
Direct N_2_O	15.15	15.15	15.15
Indirect N_2_O	13.63	13.63	13.63
Lactation+pasture feeding	4.90	4.90	4.90
Ingredient production	1.56	1.52	1.53
Transport of ingredients and feed	1.63	1.63	1.63
Electricity consumption in feed production ^1^	0.00	0.00	0.00
Farm–slaughterhouse transport	0.01	0.01	0.01
**Total**	**42.01**	**41.97**	**41.98**

^1^ TRASA’s facilities use renewable electricity, which has a 0 kg CO_2_e, as stated in the MITECO database for Adelfas Energia SL for the year 2021.

**Table 4:** The GHG-emitting activities that contribute to the total carbon footprint of the diet that includes vegetable by-products (VBP) per production phase using a physical-economic allocation (kg CO_2_e/kg meat/year). Source: Own elaboration (see Appendix A).

Conventional Diet	VBP Diet ^1^	
GHG-Emitting Activities	Emissions	
Enteric fermentation	4.17	4.46
Manure management	0.63	0.67
Direct N_2_O	9.42	15.15
Indirect N_2_O	13.33	13.63
Lactation + pasture feeding	4.59	4.90
Ingredient production	2.42	1.56
Transport of ingredients and feed	81.12	1.63
Electricity consumption in feed production	2.16	0.00
Farm–slaughterhouse transport	0.01	0.01
**Total**	**117.84**	**42.01**

^1^ The physical-economic allocation is the one used to represent the VBP diet, as it is the most complete in its estimation.

**Table 5:** The carbon footprints (in kg of CO_2_e per kg of liveweight) of cattle in the present study and other studies associated with beef production [33]. which in turn sourced the values from the references listed therein.

Study Region	Method/Boundaries/Allocation	Management	CF kg CO_2_e per kg LW
**Navarre, Spain**			
	IPCC Tier 1/cradle to slaughterhouse gate/none	Feedlot finished/study	117.8
	IPCC Tier 1/cradle to slaughterhouse gate/VBP physic-economic allocation	Feedlot finished (VBP diet)/study	41.9
**Mid-West US**			
	IPCC Tier 1 and 2/cradle to farm gate/chemical energy of co-products	Feedlot finished/study	14.8
		Pasture finished/study	19.2
**Mid-West US**			
	IPCC Tier 1 + literature/cradle to farm gate/none	Conventional cow-calf to feedlot/study	13.0
**Western Canada**			
	IPCC Tier 1 + 2/cradle to farm gate/none	Conventional cow-calf to feedlot/study	13.0
**Eastern and Western Canada**			
	IPCC Tier 1 + 2/cradle to farm gate/none	Conventional/Regional + national	15.3
			8.4
**EU-27**			
	IPCC Tier 1 + 2/cradle to farm gate + imported feed/nitrogen content of products + energetic requirements of cattle	Conventional production system specific to each EU-27 member state/national	10.4 to 13.3
**Ireland**			
	IPCC Tier 2/cradle to farm gate/mass based	Conventional suckler beef/study	13.0
**Sweden**			
	Not given/cradle to farm gate/none	Organic/study	11.6
**Charolais, France**			
	IPCC Tier 2/cradle to farm gate/not specified	Conventional/study	14.3 to 18.3
**United Kingdom**			
	Literature-based emission factors/cradle to farm gate/primarily economic	Conventional/study	8.7
		Organic and alternative/study	10.4
**European Union**			
	IPCC Tier 1/cradle to farm gate + imported feed/feed energy based	Conventional suckler beef/study	15.6
		Conventional dairy beef study/study	8.6 to 10.1
**NSW, Australia**			
	IPPC Tier 2/cradle to farm gate/none	6 different systems/study	10.1 to 12.7

## Data Availability

The data that support the findings of this study are available from the corresponding author upon reasonable request.

## Appendix A

This appendix contains data supplemental to the main text, where the baseline information and the calculator of the activities contributing to the total emissions in kg CO_2_e for each diet can be seen.

**Table A1:** Composition and origin of ingredients used in the experimental diets: Conventional diet versus vegetable by-product-based diet (VBP diet) (fresh weight) [17].

Conventional Diet ^1^			VBP Diet ^2^		
Ingredient	Percentage (%)	Origin	Ingredient	Percentage (%)	Origin
Maize	47.27	France	Maize	24.70	France
Barley	13.50	Tafalla, Navarra, Spain	Barley	15.85	Tafalla, Navarra, Spain
Straw	10.00	<30 km from Azoz, Navarra, Spain	Straw	4.07	Peralta, Navarra, Spain
Maize DDG ^3^	9.00	The Netherlands	Maize DDG ^3^	4.08	The Netherlands
Calcium carbonate	1.44	Navarra, Spain	Calcium carbonate	0.61	Navarra, Spain
Salt	0.45	Zaragoza, Spain	Salt	0.34	Zaragoza, Spain
Corrector	0.36	Tarragona, Spain	Corrector	0.27	Tarragona, Spain
Soybean meal	7.58	USA-Brasil-Argentina	Beet pulp	5.45	Miranda de Ebro, Spain
Decorticated oat Flakes	4.49	Navarra, Spain	Rapeseed meal	5.44	Germany
Palm oil	2.75	Honduras-Guatemala	Olive oil calcium soap	1.78	Madrid, Spain
Soya hulls	2.70	Argentina	Potato	8.47	<6 km from Milagro, Navarra, Spain
Sodium bicarbonate	0.32	Turkey	Carrot	7.63	
Buffer ^4^	0.14	Dinard, France	Broccoli	6.91	
			Pea	3.56	
			White bean	2.81	
			Spinach	2.35	
			Green bean	2.11	
			Pumpkin	1.36	
			Pepper	0.62	
			Aubergine	0.60	
			Red bean	0.50	
			Cauliflower	0.49	

^1^ The manufacturing plant of the Conventional diet was located in Berriozar, Navarra, Spain. ^2^ The manufacturing plant of the vegetable by-product-based diet was located in Milagro, Navarra, Spain. ^3^ DDG: dry distillers’ grains. ^4^ Buffer: Supplement for maintaining rumen pH.

**Table A2:** Enteric fermentation emissions calculated from cattle fed either a Conventional diet or a diet that includes vegetable by-products.

Enteric Fermentation	Conventional	VBP
Emission factor (kg CH_4_ calf/year)	53	53
Animals	12	12
kg CH_4_/year	640	640
kg CO_2_e/year	16,011	16,011
kg meat/year	320	299
kg CO_2_e/kg meat	4	4

**Table A3:** Manure management emissions calculated from cattle fed either a Conventional diet or a diet that includes vegetable by-products.

Manure Management	Conventional	VBP
Emission factor (kg CH_4_ calf/year)	8	8
N animals	12	12
kg CH_4_/year	96	96
kg CO_2_e/year	2400	2400
kg meat/year	320	299
kg CO_2_e/kg meat	1	1

**Table A4:** Direct N_2_O emissions calculated from cattle fed either a Conventional diet or a diet that includes vegetable by-products.

Direct N_2_O Emissions	Conventional	VBP
N animals	12	12
Average annual N excretion (kg N/animal/year)	55	53
Manure management system	Solid storage	Solid storage
Fraction of N excreted per animal and management system	37	37
Emission factor (kg N_2_O-N/kg N)	0	0
kg N_2_O/year	121	183
kg CO_2_e/year	36,140	54,425
kg meat/year	320	299
kg CO_2_e/kg meat	9	15

**Table A5:** Indirect N_2_O emissions calculated from cattle fed either a Conventional diet or a diet that includes vegetable by-products.

Indirect N_2_O Emissions	Conventional	VBP
N animals	12	12
Average annual N excretion (kg N/animal/year)	55	53
Manure management system	Solid Storage	Solid Storage
Fraction of N excreted per animal and management system	37	37
% of N in managed manure that volatilizes	45	45
Manure N lost through volatilisation of NH_3_ and Nox (kg N/year)	10,915	10,460
Emission factor (kg N_2_O-N/kg N)	0	0
kg N_2_O/year	172	164
kg CO_2_e/year	51,112	48,982
kg meat/year	320	299
kg CO_2_e/kg meat	13	14
